# Genome Sequences of Three Isolates of Fusarium verticillioides

**DOI:** 10.1128/MRA.00918-18

**Published:** 2018-08-30

**Authors:** Donald M. Gardiner

**Affiliations:** a Commonwealth Scientific and Industrial Research Organisation, Agriculture and Food, Queensland, Australia; Vanderbilt University

## Abstract

Fusarium verticillioides is an important pathogen of maize worldwide. Here, three Australian isolates of F. verticillioides, originally obtained from maize or sorghum, were sequenced using Illumina technology to expand the available genomic resources for this important pathogen.

## ANNOUNCEMENT

Fusarium verticillioides is a pathogen of maize and depending upon prevailing conditions can cause seedling blight, stalk rot, or ear rot or can even colonize endophytically (1). F. verticillioides produces a number of mycotoxins, most notably fumonisins, which are common contaminants of maize, particularly in Africa (2), and have been linked to human carcinogenesis (3).

Three F. verticillioides isolates from locations in eastern Australia were sourced from the Queensland Department of Agriculture and Fisheries Herbarium (Table 1). DNA was prepared from cultures grown in potato dextrose broth (Qiagen DNeasy plant minikit), with library generation (TruSeq Nano DNA library kit with ∼350-bp inserts) and sequencing performed by the Australian Genome Research Facility in Melbourne, Australia. Libraries were sequenced in one-eighth of an Illumina HiSeq 2500 lane (125-bp paired-end reads). Reads were trimmed using SolexaQA++ (4) (minimum Phred score, 29; minimum length, 40) before *de novo* assembly with CLC Genomics Workbench v11.0 (minimum length and similarity fractions, 95%; minimum contig size, 1,000; scaffolding disabled; mismatch cost, 2; insertion/deletion cost, 3). All genomes had similar assembly statistics (Table 1). Contigs were ordered into chromosomes based on PROmer (MUMmer package v3.07) alignment to the reference F. verticillioides genome of isolate Fv7600 (5). Other than specifying the reference and query, PROmer was executed with default settings. Based on read mapping back to the chromosomal assemblies, isolate BRIP14953 contains an ∼400-kbp duplication of one end of chromosome 2.

**TABLE 1:** Isolate details and associated features

Isolate	Source host	Sampling location^*a*^	Collection date	No. of raw read pairs (×10^6^)	Assembly length (bp)	GC content (%)	No. of contigs	*N*_50_ (bp)	Mating type	No. of protein- coding genes predicted	Locus tag	GenBank accession no.
BRIP14953	Zea mays	Walkamin, QLD	14-Mar-1977	28.6	42,454,567	48.2	1,060	97,032	MAT1-2	13,769	FVER14953	QFXM00000000
BRIP53263	Sorghum bicolor	Capella, QLD	18-Mar-2009	31.2	42,321,040	48.4	931	101,525	MAT1-1	13,195	FVER53263	QJUS00000000
BRIP53590	Zea mays	Casino, NSW	25-Feb-2010	32.1	42,219,296	48.3	1,009	105,623	MAT1-1	13,508	FVER53590	QKXB00000000

a NSW, New South Wales; QLD, Queensland.

Protein-coding genes were annotated using transcriptome sequencing (RNA-seq)-trained software. RNA-seq data, derived from isolate Fv7600, were downloaded from the Sequence Read Archive and aligned to the assemblies using TopHat (v2.1.1; intron length specified, 25 to 500 nucleotides). RNA-seq run no. SRR1810217 (6), SRR3161856, and SRR3161853 (7) were used for isolates BRIP14953, BRIP53263, and BRIP53590, respectively, creating isolate-specific alignments, albeit with some single nucleotide polymorphisms (SNPs) given the data were derived from isolate Fv7600 for training the gene prediction programs individually on each genome. CodingQuarry v2.0 (8) (BRIP14953) and BRAKER v2.1.0 (9) (BRIP53263 and BRIP53590) were used. For CodingQuarry, the TopHat RNA-seq alignment was converted to transcripts using Cufflinks (v2.2.2) specifying intron restrictions (25 to 500 nucleotides), and subsequently converted to exons (CodingQuarry GTF-to-GFF script). CodingQuarry was run with unstranded RNA-seq specified (-d). BRAKER was run with the branch point model for intron prediction (–fungus). Predictions with internal stop codons were manually corrected or removed upon inspection for all three genomes. Only single transcripts per gene were retained, keeping the highest scoring transcript (BRAKER) or transcript with the deepest RNA-seq coverage (CodingQuarry). Reciprocal best BLAST hits were used to assign homologues between the reference genome annotation (Fv7600) (10) and each isolate to maintain, where possible, consistent locus tag numbering. For example, the β-tubulin gene has the locus tag FVEG_04081 in Fv7600 and FVER14953_04081, FVER53263_04081, and FVER53590_04081 in the genomes sequenced here.

**FIG 1:**
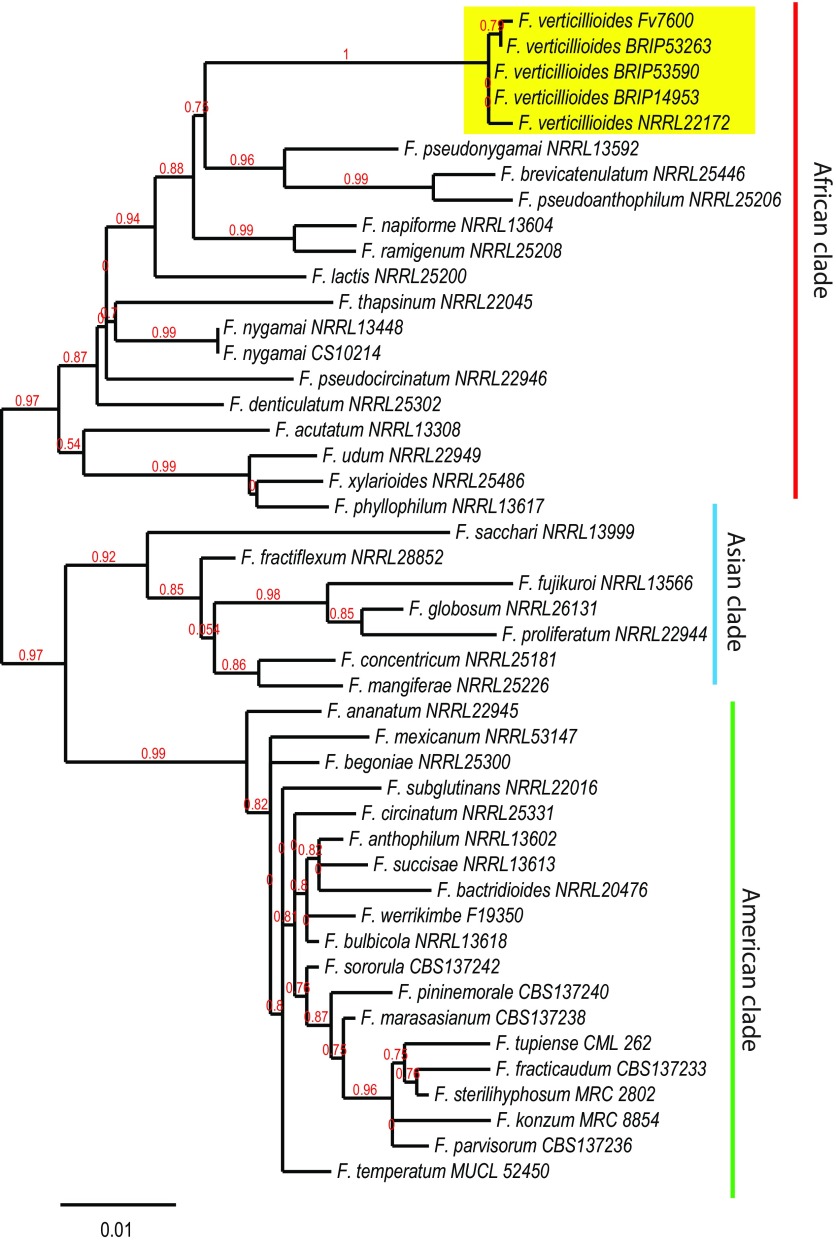
Phylogenetic placement of the three newly sequenced isolates in the Fusarium fujikuroi species complex based on combined β-tubulin and translation elongation factor 1α. The three isolates group within the F. verticillioides species (highlighted in yellow). Isolates chosen for the phylogeny were based upon named isolates found in Herron et al. (11) plus sequences extracted from the genomes of F verticillioides isolate Fv7600 and F. nygamai isolate CS10214 (GenBank accession no. MTQA00000000).

A dual-locus phylogeny using β-tubulin and translation elongation factor 1α (extracted from the assembled genomes) in reference to most of the named isolates in Herron et al. (11) identified all isolates as F. verticillioides within the Fusarium fujikuroi species complex (Fig. 1).

### Data availability.

The genome sequences and raw sequence data can be found in GenBank under the BioProject no. PRJNA437508.
